# Severe allograft rejection in an intestinal transplant patient following oral immunoglobulin treatment for chronic norovirus infection: a case report

**DOI:** 10.1002/ccr3.1493

**Published:** 2018-05-11

**Authors:** Fredrik Åberg, Johanna Savikko, Veli‐Jukka Anttila, Heikki Mäkisalo

**Affiliations:** ^1^ Transplantation and Liver Surgery Unit Helsinki University Hospital Helsinki University Helsinki Finland; ^2^ Inflammation Center Division of Infectious Diseases Helsinki University Hospital Helsinki University Helsinki Finland

**Keywords:** immunoglobulin, Intestinal transplantation, rejection

## Abstract

In an intestinal transplant patient under triple immunosuppression therapy with tacrolimus levels >10 ng/L, a 2‐day oral immunoglobulin therapy given as treatment for chronic norovirus infection was temporally closely associated with the development of severe steroid‐resistant acute graft rejection, thus suggesting that oral immunoglobulin might be able to promote a rejection response.

## Introduction

Immunoglobulin was originally used as replacement therapy in hypogammaglobulinemia patients, but has thereafter been used as off‐label treatment for a wide variety of difficult‐to‐treat autoimmune and inflammatory conditions including antibody‐mediated graft rejection after solid‐organ transplantation 1, 2.

Oral administration of immunoglobulin has been shown to be effective in infectious gastroenteritis 3, including chronic norovirus infection in intestinal transplant patients 4.

Immunoglobulin preparations comprise pooled immunoglobulin G (IgG) antibodies from sera of thousands of blood donors and exert immunomodulatory effects that are complex and incompletely understood 1. Immunoglobulin can exert both pro‐ and anti‐inflammatory activities. Although the anti‐inflammatory effects are usually desired in therapy, the consequences of pro‐inflammatory effects remain poorly studied 1. Very little is known about the local intestinal effects of oral immunoglobulin.

We report a case where the use of oral immunoglobulin for chronic norovirus infection was temporally closely associated with the development of severe steroid‐resistant acute graft rejection in a previously immunologically stable intestinal transplant patient.

## Case Report

A 34‐year‐old Caucasian male had undergone intestinal transplantation originally more than 3 years ago because of chronic intestinal pseudo‐obstruction due to familial visceral myopathy 5. The first intestinal transplant was lost due to volvulus 2 months post‐transplant. Retransplantation was performed 18 months ago. Viremic cytomegalovirus (CMV) gastroenteritis was diagnosed 1 year post‐transplant. Treatment with intravenous ganciclovir and later oral valganciclovir was successful, and CMV PCR remained negative in blood and intestinal biopsies during follow‐up. The patient also suffered another volvulus of the colon, which was successfully corrected operatively. Mild grade 1 acute rejection in the colon occurred 2 months later; the small intestine was normal. The rejection resolved quickly with intravenous steroids; follow‐up biopsies at 1 week were normal. After the rejection episode, there were nine follow‐up endoscopies showing only mild segmental erythema and distorted mucosal vascular pattern on a confined area in the colon; we speculated the previous volvulus as a cause for these mild findings. In biopsy specimens, there were no signs of rejection or inflammation, and viral samples were negative.

Norovirus gastroenteritis was diagnosed 2 months after the rejection. The patient was admitted to the hospital due to dehydration, abdominal pain, and fever. Prior to this, the patient's family members had suffered symptoms of gastroenteritis, presumably of viral origin. In our patient, gastroenteritis symptoms with watery diarrhea persisted, and the patient needed repeated episodes of hospitalization due to dehydration. Norovirus PCR from the stools remained repeatedly positive, and the norovirus infection was considered a cause for the chronic diarrhea. Endoscopy findings were unchanged. Nearly 3 months after the initial norovirus infection diagnosis, treatment with oral immunoglobulin was started. An intravenous immunoglobulin solution (Privigen^®^) was given orally at a dose of 1250 mg (25 mg/kg body weight) four times daily for 2 days (altogether eight doses). The immunoglobulin solution did not bypass the gastric barrier. Tacrolimus trough levels during the preceding 4 months had been stable above 10 ng/L (range 11.5–18.3 ng/L), and the patient had received triple immunosuppression therapy with MPA 360 mg twice daily and methylprednisolone 6 mg daily.

**Figure 1:**
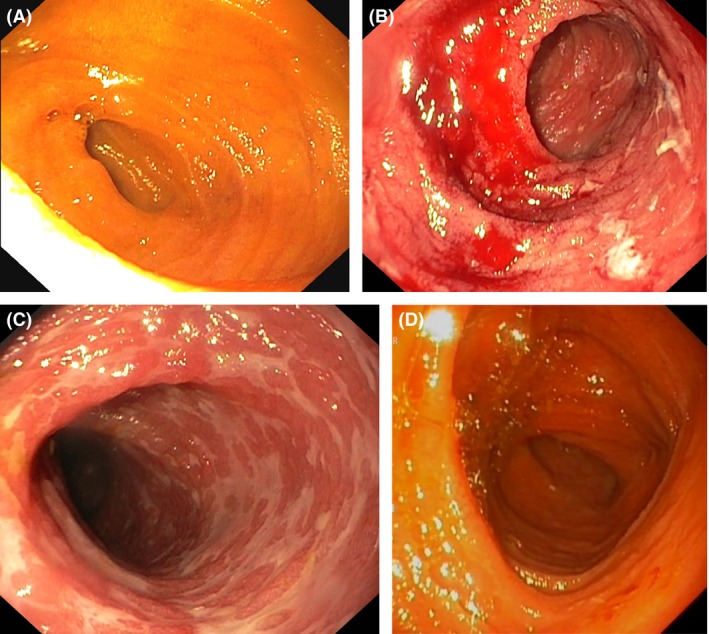
Endoscopic view of the small bowel transplant (A) before the acute rejection, (B, C) at diagnosis of acute rejection, and (D) after steroid‐ and antithymocyte globulin therapy.

**Figure 2:**
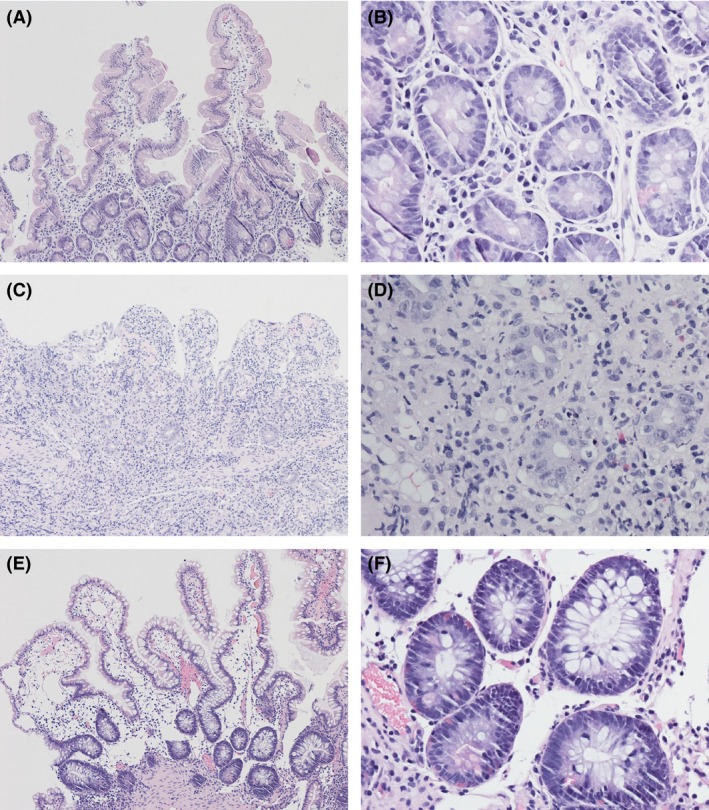
Histologic pictures of endoscopic biopsy samples from the small‐bowel graft. Before the acute rejection normal histology was demonstrated (A, magnification ×100; B, magnification ×400). At diagnosis of acute rejection moderate to severe inflammation with abundant apoptosis was seen (C, magnification ×100; D, magnification ×400). After steroid‐ and antithymocyte globulin therapy acute rejection was ameliorated and nearly normal histology was again detected (E, magnification ×100; F, magnification ×400).

Four days after the end of the immunoglobulin treatment, the patient was again admitted to hospital due to fever, increased diarrhea, and abdominal pain. Endoscopy via colostomy was performed, and severe biopsy‐proven rejection grade 2–3 was diagnosed in the small‐bowel and colon (Fig. 1 and Fig. 2). CMV and Epstein–Barr virus samples were negative. Tacrolimus trough level was 12.4 ng/L. Initial therapy was by high‐dose intravenous methylprednisolone. However, signs of rejection persisted on repeat endoscopy, and finally, a 10‐day course of intravenous antithymocyte globulin was started, whereby the acute rejection resolved both symptomatically and in graft biopsies (Fig. 1). Donor‐specific antibodies were negative. Follow‐up endoscopies showed persisting signs of colitis and distal ileitis, but these finally resolved at 3 months after starting immunoglobulin therapy, and the patient has remained rejection‐free now 10 months later.

The norovirus infection persisted, and the patient was started on nitazoxanide 500 mg twice daily, but without any clear clinical or virologic response. Bacterial, other viral, and parasitic samples were negative (Fig. 2).

## Discussion

The main message with our case is that oral immunoglobulin used to treat persistent norovirus infection could have triggered severe intestinal allograft rejection. Treatment of the chronic norovirus infection was indicated in our patient because the amounts of colostomal secretions remained high during the norovirus infection, leading to repeated dehydration episodes with acidosis and serum creatinine increases.

Possible causality between the immunoglobulin use and subsequent rejection cannot be confirmed. However, there was a very close temporal relationship between immunoglobulin use and the severe rejection, and, prior to this, nine endoscopies had been normal, immunosuppression was strong with triple therapy, and tacrolimus trough levels had been >10 ng/L. The norovirus infection was chronic and has not been reported to induce rejection. Based on this, we consider it likely that oral immunoglobulin treatment induced allograft rejection.

The immunoglobulin preparation (Privigen^®^) used in our patient comprises 98% IgG and is developed from human plasma comprising pooled fraction of serum immunoglobulin from at least 1000 donors. Little is known about side‐effects from immunoglobulin when administered orally.

Orally administered immunoglobulin has been shown to survive gastric exposure and proteolytic digestion, with the amount of intact IgG recovered in stool reportedly up to 50% 6. The effects of oral immunoglobulin are, in general, anti‐inflammatory, and there is some clinical evidence for a therapeutic benefit in, for instance, inflammatory bowel disease 3. However, published experience with the use of oral immunoglobulin in intestinal transplant patients is very scarce. Small case series have indicated that oral immunoglobulin is effective in the treatment of chronic norovirus infection in intestinal transplant patients and other solid‐organ transplant patients 4, 7. Norovirus replicates within the gut lumen, and it has been speculated that oral immunoglobulin can block viral adherence to intestinal epithelium and inhibit viral replication by forming complexes with the virus 3. Currently, there exist no effective antiviral drugs specifically for norovirus. However, nitazoxanide, an antiprotozoal drug, has shown effect in the treatment of chronic norovirus in immunosuppressed patients 8. In our case, however, we observed no clear response to nitazoxanide treatment.

The possible causal link between oral immunoglobulin use and allograft rejection, observed in our patient, is unclear. There are unique immune challenges with the intestinal allograft as the intestine harbors a high load of lymphoid cell populations, and the gut lumen is colonized by a complex microbiota. Gut microbial composition and function, the interplay between microbiota and intestinal cells, and inflammatory responses all likely affect alloresponses in intestinal transplant patients 3, 9. Specific microbial communities have been shown to promote effector and regulatory T‐cell responses 9, and changes in gut microbiota are associated with intestinal transplant rejection 10. It can be speculated that oral immunoglobulin could alter gut microbiota, interfere with local inflammatory processes, and thereby affect alloresponses 3, 9. However, direct triggering of allograft rejection by oral immunoglobulin use has, to our knowledge, not been reported. Possible residual injury to the gut from previous volvulus and/or chronic norovirus infection might also have contributed to these responses and to graft susceptibility.

In conclusion, in our intestinal transplant patient, a strong temporal relationship between the use of oral immunoglobulin for chronic norovirus infection and subsequent severe steroid‐resistant acute rejection raises concern for the possible potential of oral immunoglobulin in stimulating allograft immunologic responses. However, causality remains uncertain, and more clinical experience is needed.

## Conflicts of Interests

None declared for all authors.

## Authorship

FÅ: collected data and wrote first draft. JS: collected data and wrote the first draft. V‐JA: provided clinical insights and co‐wrote the manuscript. HM: provided clinical insights, co‐wrote the manuscript, supervised the study.
